# Tooth shell versus bone shell technique for horizontal maxillary alveolar ridge augmentation

**DOI:** 10.1186/s12903-025-05940-4

**Published:** 2025-04-25

**Authors:** Khaled Ahmed Ibrahim Awad, Mohamed Abdel-Monem Tawik, Mohamed Mohamed Hussein, Shaimaa Ahmed Abo El-Farag, Sally El Sayed Abdel Sameaa

**Affiliations:** 1 Faculty of Dentistry, Kafr El-Sheikh University, Kafr El-Sheikh, Egypt; 2 Faculty of Dentistry, Mansoura University, Mansoura, Egypt

**Keywords:** Bone shell technique, Tooth shell technique, Autogenous tooth graft, Khoury technique

## Abstract

**Background:**

This study was designed to evaluate the clinical and radiographical outcome of tooth shell for maxillary alveolar ridge augmentation as an alternative to traditional autogenous bone shell graft.

**Materials and methods:**

Twenty eight patients with one or two maxillary extracted teeth (esthetic zone) in need for horizontal bone augmentation were divided into two groups (14 patients each). Group I (control group): bone augmentation was done by using bone shell technique (BST). Group II (study group): bone augmentation was done by using tooth shell technique (TST). Implant stability was evaluated at the time of implant placement, after 4 months (loading time), and 4 months after loading.

Radiographic evaluation was made using Cone beam computed tomography (CBCT) scans before grafting (T0), immediately (T1), and 6 months post grafting (T2).

**Results:**

In this study, 30 implants were inserted in the esthetic zone. All implants were successfully osseointegrated. No statistically significant difference was found between the studied groups as regards implant stability (*P* > 0.05) assessed baseline, 4 months after implant placement, and 4 months after loading. The radiographic evaluation demonstrated a statistically significant lower median amount of graft resorption at 1 mm, 5 mm, and 10 mm & total among the study than the control group (*p* = 0.001, 0.001, 0.04 & 0.001 respectively).

**Conclusion:**

TST used for lateral ridge augmentation has excellent dimensional stability and the least amount of graft resorption.

**Trial registration:**

This study was retrospectively registered on www.clinicaltrials.gov with registration number (NCT06416605) on 16 /5 /2024.

## Background

The ideal positioning of dental implants is compromised by horizontal alveolar ridge resorption following tooth extraction, trauma, or infection [1, 2]. The split crest approach, distraction osteogenesis, guided bone regeneration, and autogenous bone transplantation are among the methods that have been suggested for widening the alveolar ridge [3, 4].

Restoring missing bone in the horizontal alveolar ridges is best accomplished with an autogenous bone graft due to its remarkable osteoinductive, osteoconductive, and osteogenetic characteristics. One drawback, though, is the frequent requirement of a donor site [5]. Due to its organic and inorganic compositional similarities to bone, dentin has recently attracted attention as a potential substitute autogenous grafting material [6–8].

Around 69% of human dentin is composed of inorganic chemicals, with organic components making up around 17.5% of the total. About 62% of alveolar bone is composed of inorganic materials, whereas 25% is organic. Osteogenetic growth factors, including BMPs, TGF-ß, and IGF-2, are mainly found in the dentin and bone organic matrix. The majority of this matrix is type I collagen, but it also contains several non-collagenous structural proteins including osteocalcin, osteonectin, phosphoprotein, and sialoprotein [9, 10].

Hydroxyapatite, ß-tricalcium phosphate, octacalcium phosphate, and amorphous calcium phosphate are the main inorganic components of dentin, similar to alveolar bone [11] These components are utilized as autogenous bone substitutes because of their excellent osteoconductive characteristics. Various animal and human clinical investigations have shown that dentin's osteoconductive and inductive characteristics can enhance bone growth at grafted defect locations [8, 12–18].

Dental implant grafts made from autogenous tooth material facilitate adequate osseointegration by participating in bone remodeling processes [19–21]. There are two types of autogenous tooth graft (AUTO-TG) can be made: as a block or a powder. There are two subtypes of block types: root-form and root-on. Since the root-form looks like a tooth root, it can be utilized to keep extraction sockets from drying out. The root-on type can be used for horizontal or vertical ridge augmentation because of its similarity in shape to a cortical block graft [17, 22, 23].

Kim et al. [24] discovered that hydroxyapatite predominates in the AUTO-TG crown, which also has a higher calcium to phosphate ratio than the remaining material. There was typically a low ratio of calcium to phosphate and modest levels of crystalline calcium phosphates in the root tissue, though. The crystallinity of enamel and dentin is what distinguishes the two. There are two types of powder-type graft materials: crown type (AUTO-TG enamel) and root type (AUTO-TG dentine). Zhang et al., [25] call this substance tooth ash. This form of crown is mostly composed of inorganic enamel that can conduct bone signals and can keep bone volume after grafting. Roots made of dentine and cementum are organic and may promote bone growth (osteoinduction) and bone loss (osteoconduction); this makes them ideal for ridge augmentations [6, 7, 26].

The tooth-shell technique is an adaptation of Khoury's bone-shell technique (BST) [27]. The use of cortical bone gained from the external oblique ridge enhances the osteoconductive properties and adds durability to cortical bone grafts in the bone-shell technique. In this case, a sturdy scaffold is formed by securely attaching the thin cortical bone shell at a distance. The resultant space is then filled with particles made of autogenous bone. Revascularization and graft regeneration are both aided by the cortical bone shell and autogenous bone particles [27]

As a result of the structural and chemical similarities of dentin and alveolar bone, equally good results can be expected for the procedure using a dentin shell and particulate dentin. If the technique is suitable for lateral ridge augmentation, postoperative discomfort could be reduced in the future in cases with teeth that cannot be preserved compared to bone block augmentation.

The aim of this study was to assess the clinical and radiographic results of using tooth shell instead of standard autologous bone shell grafts for augmentation of maxillary alveolar ridge defects.

## Materials and methods

From the outpatient clinic of the Oral Surgery Department, Faculty of Dentistry, Mansoura University, 28 patients were selected. They should have one or two maxillary teeth extracted (esthetic zone) and need horizontal bone augmentation and implant placement.

All patients were given written information regarding the treatment's advantages, risks, problems, and follow-up time in order to obtain their consent.

Under protocol number (A03030123), this study was submitted to and approved by the Dental Research Ethics Committee (Faculty of Dentistry, Mansoura University) in compliance with the seventh revision of the Helsinki Declaration in 2013. and on May 16, 2024, the study retrospectively registered in the Clinical-Trials.gov PRS (https://register.clinicaltrials.gov) with registration number (NCT06416605).

### Criteria for patient selection

#### Inclusion criteria

Patients should meet the following criteria: they should be in good physical health, have no smoking history, have one or two extracted maxillary teeth, have crestal bone widths of 4 mm or less, and be between the ages of 18 and 50. They should also have good oral hygiene.

#### Exclusion criteria

Patients undergoing radiation or chemotherapy, those abusing alcohol or drugs, those with systemic conditions that hinder bone healing (such as uncontrolled diabetes mellitus or autoimmune disease), pregnant women, those with bone disease, and those with parafunctional habits.

#### Sample size calculation

Was derived from an internal pilot study that was conducted on four samples that were not included in the full-scale investigation, and it was based on the mean difference of horizontal bone gain between the TST and BST for horizontal maxillary alveolar ridge. The sample size will be 14 in each group, using the G power program version 3.1.9.4 to compute it. This is based on a 2-tailed test with an effect size of 1.42, α error = 0.05, and power = 95.0%.

### Pre-surgical evaluation

Thorough clinical and radiographic examinations were conducted on all patients, in addition to proper history collection.

Clinical evaluation: assessing the surgery site and making sure the patient is a good fit through palpation and local visual inspection of the oral and paraoral tissues. Radiographic evaluation: utilizing CBCT.

### Patients grouping

Patients who have bone width of 4 mm or less at the alveolar crest were included in this study. Patient grouping depended on whether the patient had non-restorable molars, partially or completely impacted upper or lower third molar indicated for extraction or not. Those who had were included in group II, and others were included in group I.

CBCT was used to evaluate the morphology of the residual alveolar ridge, evaluation of the donor site in case of group I, and evaluation of non-restorable, partially or completely impacted upper or lower third molar indicated for extraction in case of group II.

### Preoperative preparation

Including scaling, patient instruction for maintaining regular teeth brushing and using regular mouthwash for one week before surgery.

As a preventive measure, the patient was advised to take 500 mg of amoxicillin every 8 h for two days before surgery (The Egyptian International Pharmaceutical Industries Co. (Emox). For one minute prior to surgery, each patient's mouth was rinsed with a mouthwash that contained 0.2% chlorhexidine gluconate (Listermix Plus, SIGMA, Egypt).

### Surgical procedure

- All procedures were performed by the same surgeon under local anesthesia in an outpatient clinic
- At the planned augmentation site, an incision was made on the middle of alveolar crest with extended gingival incisions to the adjoining teeth and two releasing incisions done vertically. After that, a mucoperiosteal elevator was used to reflect the flap buccal and palatal.

#### In group I

**Fig. 1:**
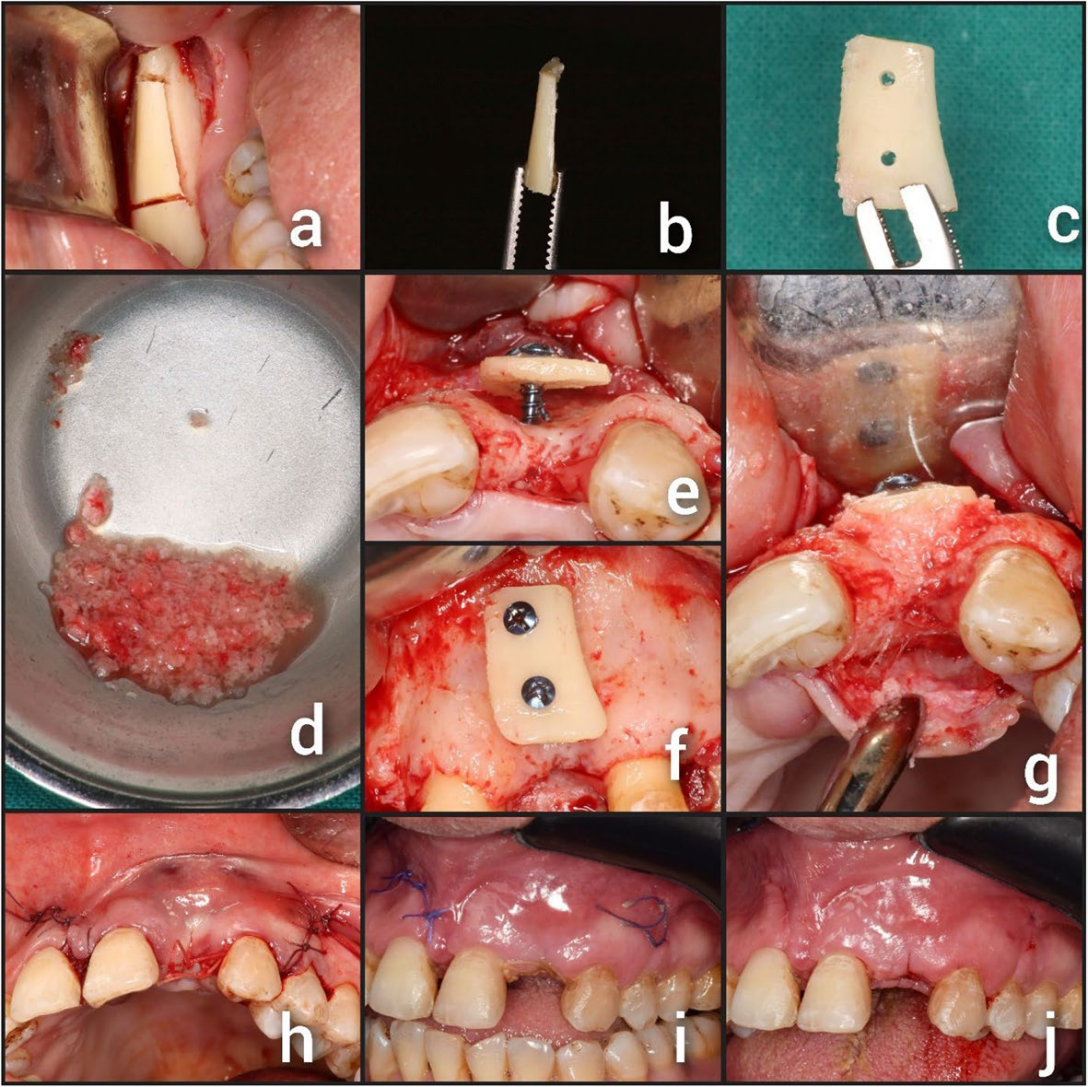
Showing a case of BST: (**a**) Using a piezoelectric device cortical bone block was harvested from the retromolar area; (**b**) The harvested bone block; (**c**) Bone shell after final preparation; (**d**) Autogenous bone chips; (**e**) Bone shell fixed in position (occlusal view); (**f**) Bone shell fixed in position (labial view); (**g**) The space between bone shell and alveolar ridge filled with autogenous bone chips; (**h**) Flap closure and suturing; (**i**) Ten days postoperative; (**j**) Suture removal

- By making a retro-molar incision and reflecting the mucosa, the external oblique ridge and ramus were exposed at the donor site.
- A piezoelectric device (ACTEON: Manufactured by SATELEC®, France) was used to cut autogenous cortical bone block from the retromolar region Fig. 1 (a).
- The graft was obtained with the help of a small bone chisel and hammer (Fig. 1 (b)). The autogenous bone block was divided into two cortical shells, each measuring 1–1.5 mm, by cutting it along its longitudinal axis with a surgical disc (Fig. 1 (c)).
- Until the recipient site preparation was completed, the two cortical bone shells were kept in saline solution.
- A fine surgical drill was used to fenestrate the recipient site multiple times for better blood flow to the site of augmentation.
- One of the cortical bone shells was prepared to the recommended thickness (1–1.5) mm and the other bone shell was crushed to small particles by using a bone crusher.
- An autogenous chip maker (ACM) drill was used to obtain more autogenous bone chips from the donor surgical site Fig. 1 (d).
- Using 2 microscrews (Self-tapping fixation screws 7, 9, 11 mm in length were selected according to the drill depth. Dual Top Anchor, Jeil Medical Corporation, Korea) to secure the produced cortical shell at a distance from the residual bone, it was used to restore the alveolar ridge. The bony borders were then smoothed Fig. 1 (e, f).
- To fill the space between the alveolar bone and the fixed bone shell, autogenous bone chips were used Fig. 1 (g).

#### In group II

**Fig. 2:**
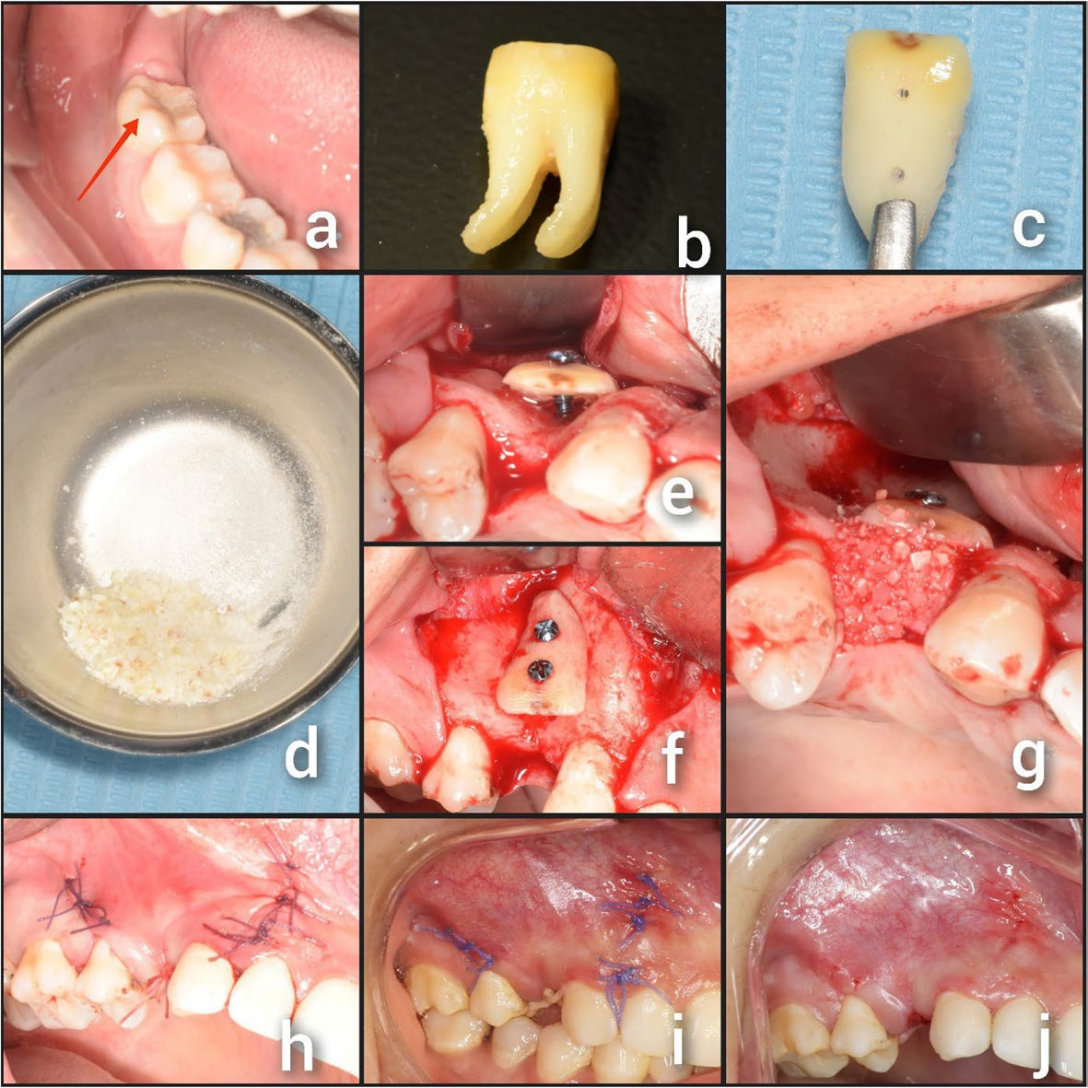
Showing a case of TST: (**a**) Tooth indicated for extraction; (**b**) The extracted tooth after removal of enamel and cementum (**c**) Tooth shell after final preparation; (**d**) The remaining portion of the extracted tooth after grinding; (**e**) Tooth shell fixed in position (occlusal view); (**f**) tooth shell fixed in position (labial view); (**g**) The space between tooth shell and alveolar ridge filled with autogenous dentin particles; (**h**) Flap closure and suturing; (**i**) Ten days postoperative; (**j**) Suture removal

- A mechanical cleaning was performed on the tooth that would be utilized for the augmentation, including the removal of debris and periodontal ligament, right after extraction. A coarse diamond bur was used under water cooling to remove any cementum, enamel, and restorations Fig. 2 (a, b)
- Then, a tooth slice was cut longitudinally by a diamond disk. A tooth shell (about 1–1.5 mm thick) was created using a diamond bur Fig. 2 (c).
- The remaining tooth structure was ground into particles ranging from 300 to 1200 µm using a sterilized grinder [28] Fig. 2 (d).
- By soaking the tooth graft in a solution of 10% EDTA for three minutes, we were able to partially demineralize the dentin, it enabled the release of active growth factors and the exposure of the collagen fiber network. Afterwards, a buffered saline solution was used to clean the material once more.
- A fine surgical drill was used to fenestrate the recipient site multiple times, improving the blood flow to the augmentation site.
- Microscrews (Self-tapping fixation screws 7, 9, 11 mm in length were selected according to the drill depth. Dual Top Anchor, Jeil Medical Corporation, Korea) were used to secure the tooth's shell laterally to the alveolar bone defect (Fig. 2 (e, f)). The autogenous dentin particles were then placed into the space between the alveolar bone and the fixed tooth shell Fig. 2 (g).

### For both groups

No membrane was used over the augmented bone.

In order to establish the mucoperiosteal flaps without tension, periosteal releasing incisions were made to advance them coronally. The flaps were then repositioned and sutured using a 4–0 vicryl suture (Figs. 1 (h) and 2 (h)).

### Postoperative care

The antibiotic Amoxicillin 1 g was taken orally once every 12 h for a duration of seven days. 50 mg tablets of Diclofenac Potassium (Oflam, Mepha Pharma Egypt S.A.E.) were given 3 times per day as an anti-inflammatory and non-steroidal analgesic. Using Chlorohexidine HCl (0.12%) (Hexitol, the Arab Drug Company, Cairo, A.R.E.), patients were instructed to keep their mouths clean and to refrain from chewing solid foods.

The sutures were removed after ten days Figs. 1 (i, j) and 2 (i, j).

**Fig. 3:**
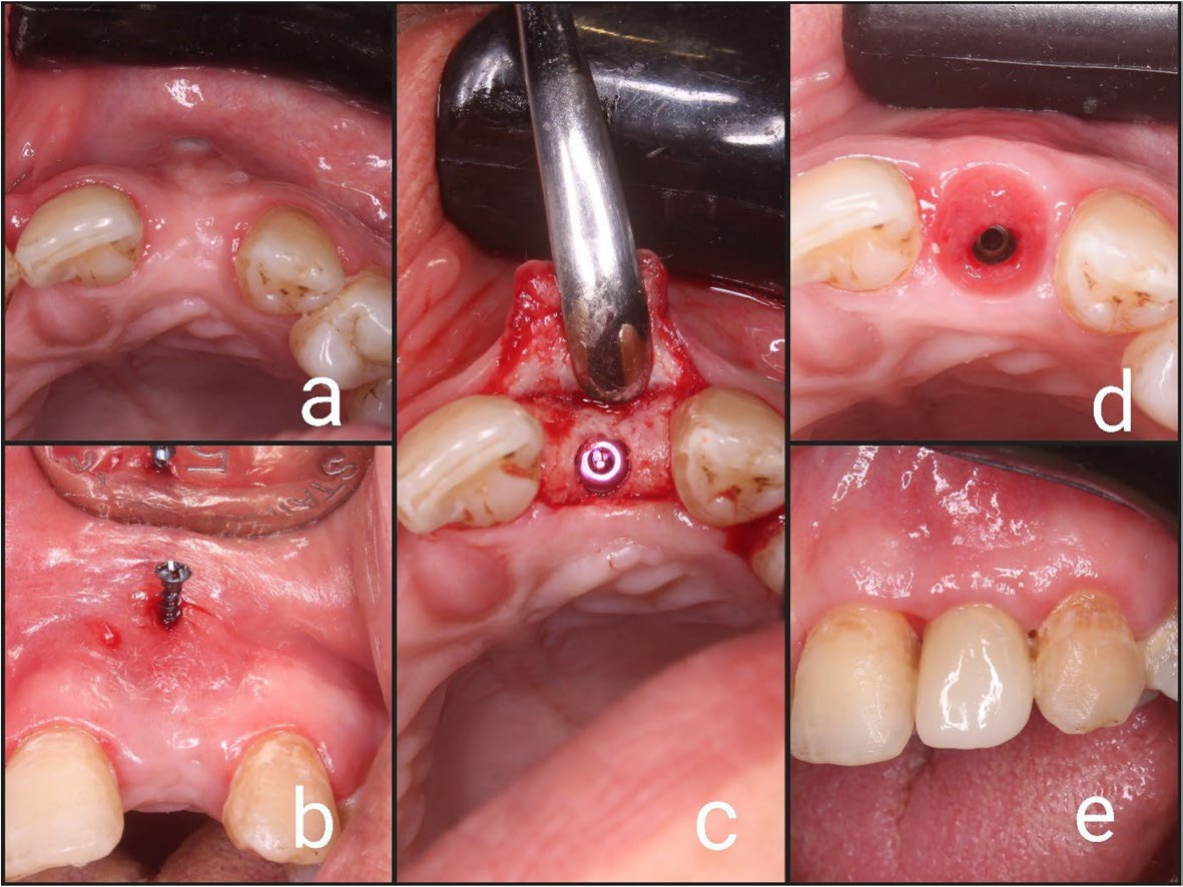
Showing follow up of the case of BST: (**a**) Surgical site after 6 months; (**b**) Removal of microscrew; (**c**) Implant placement; (**d**) Emergence profile; (**e**) Final prosthesis

**Fig. 4:**
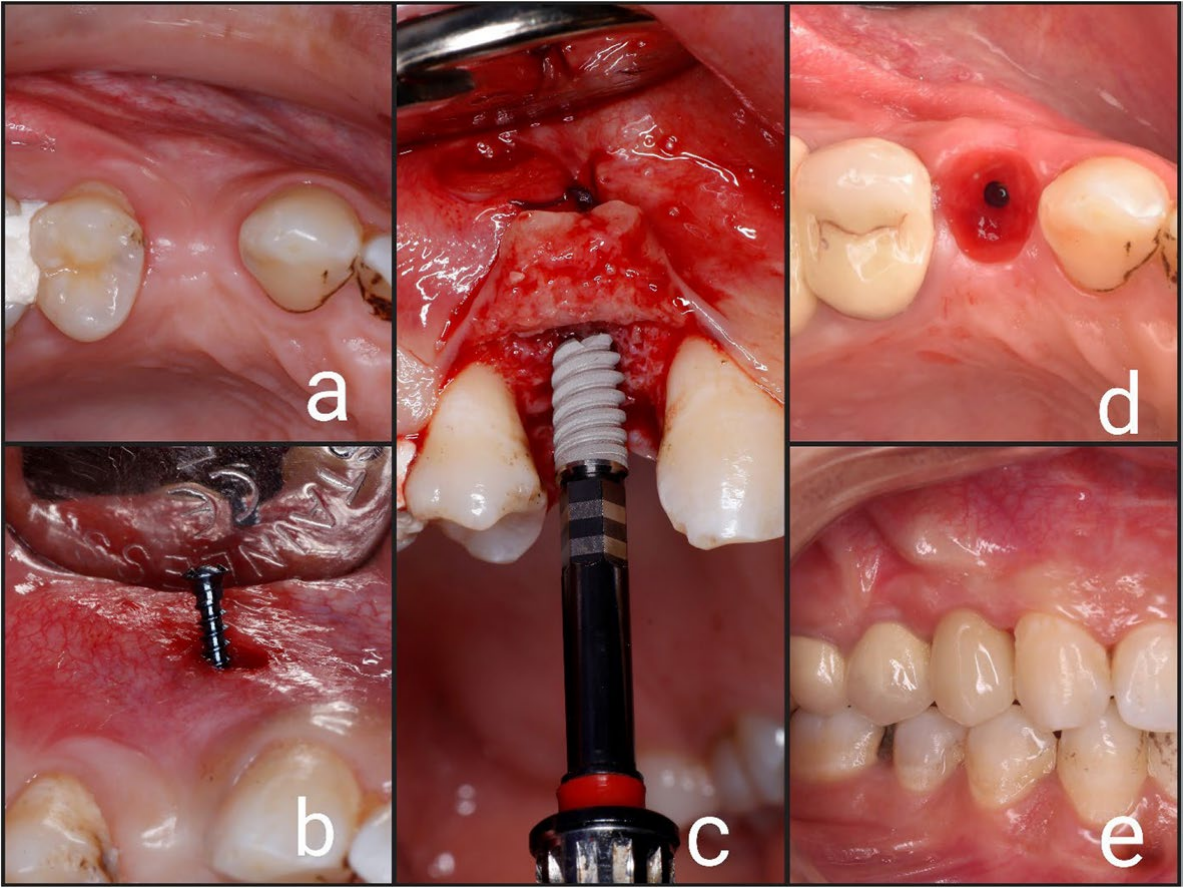
Showing follow up of the case of TST: (**a**) Surgical site after 6 months; (**b**) Removal of microscrews; (**c**) Implant placement; (**d**) Emergence profile; (**e**) Final prosthesis

After 6 months, the osteosynthesis screws were removed through a small incision over the screws Figs. 3 (a, b) and 4 (a, b).

A crestal incision was made, the implant site was prepared (Conventional, 2 pieces, screw type dental implant was used, Vitronex, Italy) and the dental implant was inserted in a prosthetically driven position. The same implant size of 3.7 mm diameter and 13 mm length was used for all cases Figs. 3 (c) and 4 (c).

A standard reentry procedure was carried out 4 months after implant placement, the implant cover screw was exposed and removed, a healing abutment was then placed for 2 weeks to get a good soft tissue contouring Figs. 3 (d) and 4 (d).

### Prosthetic phase

After making an impression using impression post and implant analog, a working cast was fabricated and the final restoration was made from zirconia and screw retained Figs. 3 (e) and 4 (e).

### Evaluation

#### Clinical evaluation

- For the first month following surgery, patients were monitored weekly; thereafter, they were monitored monthly for the remaining six months until implantation.
- The patients were assessed for mucosal healing, pain, edema, and hematoma at the donor and recipient sites.

### Implant stability

Primary implant stability was measured using Ostell at the time of implant insertion as a baseline reading of implant stability quotient 1 (ISQ1). Ostell (ISQ) values were also used to evaluate secondary implant stability 4 months after implant insertion (ISQ 2) and 4 months after loading (ISQ 3). The measurement site of ISQ was only from the buccal side.

### Radiographic evaluation

CBCT scans were taken at three stages; T0 (before grafting), T1 (immediately after grafting), T3 (6 months post grafting) for linear measurements of alveolar bone width.

pre and postoperative CBCT scans were imported to "fusion" module of Ondemand3D app software (v.1.0.10.7510, Cybermed, Korea) voxel based superimposition was done to realign post dicom dataset "secondary" as mentioned by software to the pre "primary" one so, reference lines representing axial, coronal and sagittal sections are synchronized.

**Fig. 5:**
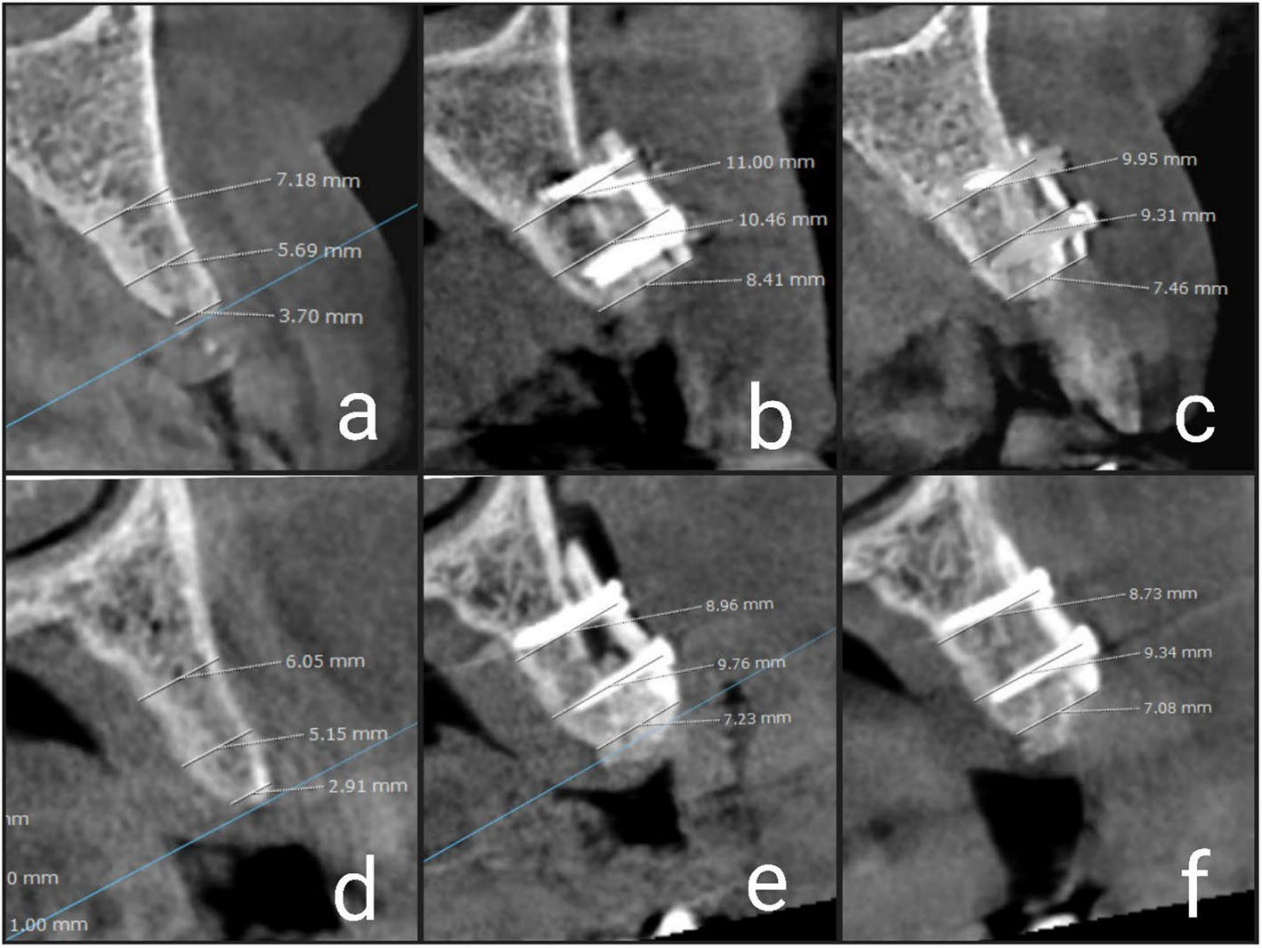
Showing radiographic evaluation: BST (**a**-**c**); (**a**) Preoperative; (**b**) Immediate postoperative; (**c**) After 6 months. TST (**d**-**f**); (**d**) Preoperative; (**e**) Immediate postoperative; (**f**) After 6 months

Using the same fixed points for each measurement, we were able to determine the buccolingual width at three distinct levels: 1 mm below the crest, 5 mm from the bone crest, and 10 mm from the bone crest. (Fig. 5) In order to do statistical analysis, the measurements were tabulated.

$$\mathrm{Amount}\;\mathrm{of}\;\mathrm{bone}\;\mathrm{gain}\;(\mathrm{BG}=\mathrm T2-\mathrm T0)$$

$$\mathrm{Amount}\;\mathrm{of}\;\mathrm{bone}\;\mathrm{resorption}\;(\mathrm{BR})=\mathrm T1-\mathrm T2$$

### Statistical analysis

Analysis and interpretation of data: SPSS software, version 25, was used for data analysis (SPSS Inc., PASW statistics for Windows version 25). Springfield, Illinois: SPSS, Inc. Qualitative data was represented by percentages and numbers. After determining if the data was normally distributed using the Shapiro–Wilk test, the quantitative data was reported using the mean ± standard deviation and non normally distributed data as median (min–max). We used a significance level of 0.05 to evaluate the outcomes. When comparing qualitative data between groups, suitable tests such as Chi-square and Fisher exact were utilized. Student t test and Mann Whitney U test were employed to compare the two separate groups for normally and non normally distributed data respectively.

## Results

Thirty dental implants were used to replace extracted front and premolar teeth (esthetic zone) in 28 patients (16 females and 12 males) in this study. The procedure includes horizontal ridge augmentation and delayed implant insertion. With ages ranging from twenty to forty-five, the average was thirty. A total of fifteen maxillary incisors, ten lateral incisors, two canines, and three first premolars were replaced.

In the majority of instances, there were no notable problems during or after the operation, and no indications of infection were detected clinically.

There were no reported sensory impairments along the path of the inferior alveolar nerve in the control group. The control group experienced more postoperative edema than study group as all extracted teeth were simply extracted.

Throughout the entire follow-up period, there was a perfect 100% survival rate, indicating that all implants were osseointegrated effectively.

### Implant stability

There was no statistically significant difference between the studied groups as regards implant stability (*P* > 0.05) assessed baseline, 4 months after implant placement, and 4 months after loading. However high stability was detected among the study group at the last follow-up (all cases show high stability) Table 1.

**Table 1:** Comparison of implant stability grading between the studied groups

Implant stability	Control *n* = 14	Study *n* = 14	Test of significance
**Baseline**			
High stability	0	0	
Medium stability	2(14.3%)	2(14.3%)	
Low stability	12(85.7%)	12(85.7%)	*P* = 1.0
**4 months after implant insertion**			
High stability	4(28.6%)	2(14.3%)	FET = 0.848
Medium stability	10(71.4%)	12(85.7%)	*P* = 0.648
Low stability	0	0	
**4 months after loading**			
High stability	12(85.7%)	14(100%)	FET = 2.15
Medium stability	2(14.3%)	0	*P* = 0.481
Low stability	0	0	

Used test: Fisher exact test

### Radiographic analysis

No statistically significant difference was found between the studied groups as regards ridge width assessed at T0, T1, and T2 except for ridge width of 1 mm at T2 (*p* = 0.001), 5 mm (*p* = 0.027) and ridge width total at T2 (*p* = 0.001), with higher mean ridge width is detected for study than control group. For the study group; there was a statistically significant increase in ridge width from T0 to T1 and a decrease at T2. Similarly for the control group; there was a statistically significant increase and then decrease at different measurements 1 mm, 5 mm, 10 mm, and total Table 2.

**Table 2:** Comparison of ridge width between study and control groups

**Ridge width**		**Control** ***n* = 14**	**Study** ***n* = 14**	**Test of significance**
**T0**	1 mm	3.27 ± 0.67	3.04 ± 0.44	*t* = 1.03 *p* = 0.313
	5 mm	5.48 ± 1.08	5.45 ± 0.65	*t* = 0.072 *p* = 0.943
	10 mm	7.51 ± 1.54	6.93 ± 1.46	*t* = 1.02 *p* = 0.317
	Total	5.41 ± 0.84	5.14 ± 0.79	*t* = 0.884 *p* = 0.385
**T1**	1 mm	8.11 ± 0.38	7.96 ± 0.66	*t* = 0.715 *p* = 0.481
	5 mm	10.47 ± 0.74	10.09 ± 0.79	*t* = 1.29 *p* = 0.206
	10 mm	11.03 ± 1.72	10.69 ± 1.32	*t* = 0.594 *p* = 0.558
	Total	9.87 ± 0.84	9.58 ± 0.81	*t* = 0.928 *p* = 0.362
**T2**	1 mm	4.79 ± 0.77	7.49 ± 0.74	*t* = 9.44 *p* = 0.001*
	5 mm	8.88 ± 0.90	9.69 ± 0.92	*t* = 2.35 *p* = 0.027*
	10 mm	10.27 ± 1.39	10.36 ± 1.41	*t* = 0.178 *p* = 0.860
	Total	7.98 ± 0.88	9.18 ± 0.87	*t* = 3.62 *p* = 0.001*
	for 1 mm	P1 = 0.001* P2 = 0.001* P3 = 0.001*	P1 = 0.001* P2 = 0.001* P3 = 0.001*	
	for 5 mm	P1 = 0.001* P2 = 0.001* P3 = 0.001*	P1 = 0.001* P2 = 0.001* P3 = 0.001*	
	for 10 mm	P1 = 0.001* P2 = 0.001* P3 = 0.001*	P1 = 0.001* P2 = 0.001* P3 = 0.001*	

*t* Student *t* test P1: Comparison between T0&T1, P2: Difference between T0&T2, P3: Difference between T1&T2

### Amount of bone gain

No statistically significant difference was found between the studied groups regarding horizontal bone gain at 5 mm and 10 mm. However, a statistically significant higher median horizontal bone gain at 1 mm among study than control group (4.21 and 1.38, respectively, *p* = 0.001). Also; a statistically significant higher median total horizontal bone gain among study than control group (3.78 and 2.89, respectively, *p* = 0.001). Table 3

**Table 3:** Comparison of horizontal bone gain among the studied groups

		**Control** ***n* = 14**	**Study** ***n* = 14**	**Test of significance**
**Horizontal Bone Gain (BG**	1 mm	1.38 (0.7–2.63)	4.21 (3.54–5.47)	*z* = 4.51 *p* = 0.001*
	5 mm	4.0 (-0.22, 5.05)	4.19 (3.48–4.86)	*z* = 1.38 *p* = 0.167
	10 mm	3.53 (0.01–4.45)	3.49 (2.69–4.19)	*z* = 0.092 *p* = 0.927
	Total	2.89 (0.3–3.69)	3.78 (3.67–4.82)	z = 4.33 *p* = 0.001*

Z:Mann Whitney U test *statistically significant

data are expressed as median (min–max)

### Amount of bone resorption

Compared to the control group, the experimental group exhibited significantly decreased median amounts of graft resorption at 1 mm, 5 mm, and 10 mm and total (*p* = 0.001, 0.001, 0.04 & 0.001, respectively) Table 4.

**Table 4:** Comparison of amount of graft resorption among the studied groups

		**Control** ***n* = 14**	**Study** ***n* = 14**	**Test of significance**
**Amount of Graft Resorption**	1 mm	3.15 (2.41–4.06)	0.35 (0.26–0.86)	*z* = 4.51 *p* = 0.001*
	5 mm	1.24 (0.43–2.99)	0.40 (0.20–0.70)	*z* = 4.14 *p* = 0.001*
	10 mm	0.68 (0.01–1.99)	0.22 (0.07–0.75)	*z* = 1.91 *p* = 0.04*
	Total	1.69 (1.45–2.52)	0.41 (0.20–0.60)	*z* = 4.51 *p* = 0.001*

Z:Mann Whitney U test *statistically significant

data are expressed as median (min–max)

## Discussion

Due to the disparity between the implant's diameter and the horizontal dimension of the alveolar ridge, a narrowing of the ridge poses a substantial challenge to the successful placement of dental implants. The final prosthesis may not be functionally or aesthetically acceptable due to intraoperative problems, improper placement, inadequate bone support from the buccal and palatal or lingual walls around the implant, fenestration of the cortical wall, and other issues [29–34].

To fulfill implantation criteria, there are several therapeutic methods for repairing horizontally deficient alveolar ridges. These methods include ridge splitting or expansion, distraction osteogenesis (DO), bone block grafts, guided bone regeneration (GBR), osteoplasty to enhance the width, narrower diameter implants, and combinations of these and other methods [4, 35–40].

The gold standard for enhancing severe horizontal ridge resorption has been autogenous onlay block grafting. For ridge abnormalities that could not be addressed using extra-oral donor sites, Khoury developed a bone shell approach that utilized mandibular chin or ramus blocks as an alternative. Using BST has shown minimal amount of graft resorption and good vascularization to the graft when compared with the conventional autogenous bone block graft [27, 41, 42].

The Khoury BST provides a method for the predictable reconstruction of even the most complicated alveolar ridge abnormalities [27]. When compared to other methods of alveolar ridge repair, BST offers a high success rate with a low complication rate [27, 43, 44]. The BST has been used as the perfect control group due to its similarities to the TST and the reasons stated above. However, there are risks to this method, particularly in the donor area, where problems like infections or inferior alveolar nerve damage might occur.

The TST done in our study was a modification of the technique using tooth dentin graft rather than autogenous bone grafts, in which taking autogenous bone grafts from retromolar area or other donor sites have been avoided. Because of its structural and chemical similarities to bone, autogenous dentin has demonstrated to be an effective bone replacement material with favorable biological characteristics and minimal graft resorption, according to numerous studies [45, 46].

Nevertheless, it has demonstrated that autogenous dentin plays a role in bone remodeling and gradually a newly formed bone has been deposited through replacement resorption. This process is more uniform than bone transplants, but it does leave behind some remains of the tooth material [47].

There are two types of dentin grafts: mineralized and demineralized. The benefit of the demineralization therapy is that it releases growth factors and reveals the collagen matrix, increasing the body's ability for regeneration [48]. However, a loss in osteoconductivity and a relative degradation of growth hormones are some of the disadvantages of this procedure. In contrast, the mineralised version retains both the organic and inorganic. As a result, the removed tooth once considered a weakened organ that was no longer in use can be used as an inexpensive bone substitute [11].

Cinar et al. Similar results were obtained when quick implantation in a new extraction socket was combined with either autogenous mineralized dentin graft or xenogeneic graft material augmentation. Both materials were favorable and helpful. According to the study's clinical and radiological examination, the autogenous dentin graft demonstrated effectiveness that was about on par with the xenogeneic [49].

In this study, a 10% EDTA solution was used to partially demineralize the dentin [14]. This process can induce new bone development and replacement resorption by exposing the networks of collagen fibers and releasing many growth factors as BMPs. Conversely, because the collagen network is hydrolyzed by enzymes, fully demineralized dentin is resorbed before new bone can be produced [48]. The TST has a lower resorption rate when compared to the BST, this was suggested by the somewhat demarkable bone shell and the clear sight of the tooth shell in the CBCT scan.

In the present study, healing of TST was excellent without any complications or infection during the healing period of the graft and the newly formed bone show good vascularity similar to normal surrounding bone. This is in line with the findings of Schwarz et al., who demonstrated during implant placement clinical evaluations that grafted bone exhibits bleeding properties comparable to the natural surrounding bone [46]. Additionally, AUTO-TG demonstrated remarkable biocompatibility in long-term clinical trials undertaken by Lee & Kim et al. [50, 51]. The results show that AUTO-TG can withstand infections and heals well, even when wounds are slightly open. A further study by Kim et al. showed that AUTO-TG was replaced by a high-quality bone gradually by a combination of osteoinduction and osteoconduction after a period of slow resorption [50].

There were no major problems (graft and/or implant loss) with TST and BST in this study. There was a perfect record of implant survival. The BST harvesting process did not result in any nerve damage causing either transitory or persistent paraesthesia. Previous studies have shown that both TST and BST have complication rates that are within acceptable ranges [5, 27, 43, 45, 46, 52–54].

There was no dehiscence reported within the TST group that composed of 14 patients. All patients show excellent healing during follow up period. In contrast with our study, Lee et al. [51] reported that, two out of nine patients in their case series of autogenous tooth grafting had wound dehiscence, the anterior mandible ridges of both individuals were grafted horizontally. Wound dehiscence was also documented in a single patient out of twelve patients in the case series by Kim et al. [50]. Ultimately, both authors achieved satisfactory outcomes with minimal graft material loss by cautiously managing dehiscence with antiseptic dressings containing chlorhexidine.

Schwarz et al. [45] described using a full tooth root as an augmentation material, although the diameter of the implant can't be wider than the root itself. Similar to Khoury's bone block grafting method [27], the tooth shell procedure outlined here can enhance a bigger horizontal loss. Revascularization and regeneration are anticipated to have greater outcomes with particle dentin in the space between the dentin shell and the bone compared to a technique utilizing solid dentin blocks [45].

In our study, horizontal alveolar ridge augmentation was done first and after 6 months of healing dental implants were inserted. In contrast Korsch et al., retrospective study involved 28 patients (15 females and 13 males) with a total of 34 areas and 38 implants who underwent tooth-shell technique (TST) lateral ridge defect restoration using autogenous dentin slices. In a control group of 31 patients (16 females and 15 males), they utilized the bone-shell technique (BST) on autogenous bone according to Khoury's description. This approach involved 32 locations and 41 implants. Because they were all inserted simultaneously during augmentation, all of the implants in both groups had entirely osseointegrated [55].

Predicting the success of implant osseointegration and the choice of loading technique has been done using implant stability quotient (ISQ). The success rate of implants has been found to increase with an elevated ISQ [56]. In this study, implant stability was measured at the time of implant placement, at time of loading (4 months after implant placement), and again after 4 months of loading.

Results from the ISQ measured during implant placement were consistent with previous research [57, 58]. Implant stability improved significantly over the follow-up period, which may be attributable to deeper osseointegration and the grafted bone's slow but steady growth.

Regarding implant stability, which was evaluated at the implant placement time, four months after implant placement, and four months after loading, no statistically significant difference was found between the groups that were tested (*P* > 0.05). Consistent with Korsch et al., all implants had ISQ values above 60, ranging from 61 to 89. Both BST and TST had average ISQ values of 74.7 and 73.3, respectively. None of the categories differed significantly from one another [55].

Statistical examination of CBCT radiographs revealed that the experimental group had significantly less graft resorption at 1 mm, 5 mm, and 10 mm total compared to the control group (*p* = 0.001, 0.001, 0.04 & 0.001, respectively). Research by Kim et al. [6], and Parvini et al. [59], has shown similar results. Two factors contributed to this discovery. Firstly, during basal ankylosis, new structures involving fibrous tissue and woven bone quickly emerge on the basal side, which initiates the resorption of AUTO-TG [45, 60]. In addition to facilitating the attachment of collagen fibers, dentin's extreme rigidity inhibits root absorption at the surface [45, 61]. On the flip side, due to the presence of weak vascular tissue and a large number of non-vital bone regions, AUTO-BG without barrier membrane protection may speed up absorption [62].

On the other hand, korsch et al. discovered that there was no statistically significant differences found among the study groups at any of the three levels of Statistical analysis when they evaluated the CBCTs after 3 months of ridge augmentation and simultaneous implant placement [55].

This study has some limitations such as a small sample size, the follow up period was relatively short, no membrane used at the augmentation area, soft tissue factors such as the width of the keratinized mucosa, the biotype of the gingival, and the position of gingival zenith were not assessed. It is advised to conduct additional research using comparison x-rays, histological analyses and lengthier observation times.

## Conclusion

However the limitations, this study proved that lateral alveolar ridge augmentation using the tooth shell approach is a safe and dependable grafting procedure for lateral alveolar ridge augmentation with predictable results. Due to the avoidance of a second intervention for the harvesting of autogenous bone, the burden on the patient can be minimized.

## Data Availability

The sets of data that have been utilized and/or analyzed for this study can be provided by the corresponding author upon reasonable request.

## Abbreviations

BST: Bone shell technique
TST: Tooth shell technique
CBCT: Cone-beam computed tomography
IRB: The Institutional Review Board
AUTO-TG: Autogenous tooth graft
ACM: Autogenous chip maker
ISQ: Implant stability quotient
BG: Bone gain
BR: Bone resorption
DO: Distraction osteogenesis
GBR: Guided bone regeneration
AUTO-BG: Autogenous bone graft
BMPs: Bone morphogenetic proteins
